# The influence of tumor necrosis factor-α on the tumorigenic *Wnt*-signaling pathway in human mammary tissue from obese women

**DOI:** 10.18632/oncotarget.16632

**Published:** 2017-03-28

**Authors:** Agathe Roubert, Kelly Gregory, Yuyang Li, Anna C. Pfalzer, Jinchao Li, Sallie S. Schneider, Richard J. Wood, Zhenhua Liu

**Affiliations:** ^1^ Nutrition and Cancer Prevention Laboratory, School of Public Health and Health Sciences, University of Massachusetts, Amherst, MA, USA; ^2^ Pioneer Valley Life Sciences Institute, Baystate Medical Center, Springfield, MA, USA; ^3^ Department of Surgery, Shandong Provincial Hospital, Shandong University, Jinan, Shandong, China; ^4^ Jean Mayer USDA Human Nutrition Research Center on Aging, Tufts University, Boston, MA

**Keywords:** obesity, inflammation, tumor necrosis factor-α, Wnt pathway, breast cancer

## Abstract

Epidemiological studies have convincingly suggested that obesity is an important risk factor for postmenopausal breast cancer, but the mechanisms responsible for this relationship are still not fully understood. We hypothesize that obesity creates a low-grade inflammatory microenvironment, which stimulates *Wnt*-signaling and thereby promotes the development of breast cancer. To test this hypothesis, we evaluated the correlations between expression of multiple inflammatory cytokines and *Wnt* pathway downstream genes in mammary tissues from women (age ≥ 50) undergoing reduction mammoplasty. Moreover, we specifically examined the role of tumor necrosis factor-α (TNF-α), an important proinflammatory cytokine associated with obesity and a possible modulator of the *Wnt* pathway. The regulatory effects of TNF-α on *Wnt* pathway targets were measured in an *ex vivo* culture of breast tissue treated with anti-TNF-α antibody or TNF-α recombinant protein. We found that BMI was positively associated with the secretion of inflammatory cytokines IL-1β, IL-6 and TNF-α, all of which were negatively correlated with the expression of *SFRP1*. The transcriptional expression of *Wnt*-signaling targets, *AXIN2* and *CYCLIN D1*, were higher in mammary tissue from women with BMI ≥ 30 compared to those with BMI < 30. Our *ex vivo* work confirmed that TNF-α is causally linked to the up-regulation of active β-CATENIN, a key component in the *Wnt* pathway, and several *Wnt*-signaling target genes (*i.e. CYCLIN D1, AXIN2, P53* and *COX-2*). Collectively, these findings indicate that obesity-driven inflammation elevates *Wnt*-signaling in mammary tissue and thereby creates a microenvironment conducive to the development of breast cancer.

## INTRODUCTION

Epidemiological studies have convincingly suggested obesity is a critical risk factor for postmenopausal breast cancer with a 12% increase of risk for every 5 kg/m^2^ of BMI [1, 2]. With 66% of American being overweight or obese [3], the public health implications of obesity on postmenopausal breast cancer risk are significant. However, the molecular mechanism(s) linking obesity to an increased risk of postmenopausal breast cancer remain incompletely understood. The recognition of obesity as a state of chronic low-grade inflammation supports a potential role of inflammatory cytokines in obesity-associated breast cancer [4, 5].

In addition to its lipid-storing capacity, adipose tissue possesses important endocrine functions that can stimulate inflammatory responses, including the elevation of macrophages and T-helper cells. These immune cells, as well as enlarged adipocytes, are responsible for the production of pro-inflammatory cytokines such as IFN-γ, IL-1β, IL-6, IL-8, and TNF-α [6–8]. As such, adipose tissue surrounding mammary epithelial cells can invoke a potent inflammatory microenvironment that may initiate pro-carcinogenic molecular and physiological changes and promote breast tumorigenesis [9, 10]. In addition, since elevated levels of serum inflammatory cytokines are observed in obese individuals [4], inflammatory cytokines produced by adipose tissue in other areas of the body could also circulate to the breast and affect the mammary microenvironment.

The *Wnt*/β-catenin cell signaling pathway is a critical regulatory pathway of tumorigenesis that controls cell proliferation, migration, and differentiation [11, 12]. In colorectal cancer, genetic mutation of Adenomatous Polyposis Coli (*APC)* in the *Wnt*-signaling cascade is a major contributing factor for familial colorectal cancers [13], but it is typically not the primary mechanism associated with breast cancer. It has been demonstrated that only 6% of breast tumors contain *APC* mutations and mutations in the β-catenin gene in breast cancer are very rare [14, 15], despite noted *Wnt*-signaling abnormalities associated with 60% breast tumors [16, 17]. Therefore, inappropriate activation of the *Wnt*-signaling pathway may be due to mediators other than the mutation of *Apc* in the development of breast cancer.

To date, there are limited empirical data to delineate how obesity-promoted inflammation activates the pro-tumorigenic *Wnt*-signaling, particularly in mammary tissue. Recently, data from our laboratory [18] indicate that, in obese mice, TNF-α may result in elevated phosphorylation of *GSK3β, a* key component of the *Wnt* pathway in the colon, and this association is also manifested in an *in vivo* model of gastral cell lines [19]. In the present human studies, we examined the association of obesity with inflammatory cytokines and the expression of *Wnt* target genes in mammary tissue from women with a variety of BMI. We further demonstrated the causal role of TNF-α in the regulation of *Wnt* target gene expression in an explant culture of mammary tissue treated with anti-TNF-α antibody or TNF-α recombinant protein.

## RESULTS

### Anthropometric characteristics of the subjects

As described in the following Materials and Methods section, due to the fact that subjects who underwent reduction mammoplasty were mainly obese, subjects were categorized into 2 groups: BMI ≥ 30 group and BMI < 30 group. In the first association study (*Exp. I*, Supplementary Table 1), of the 26 subjects, 10 subjects had BMI < 30 with an average of 25.6 kg/m^2^ (range: 21 – 29), and 16 subjects had BMI ≥ 30 with an average of 36.0 kg/m^2^ (range 30–48). There were no differences in age between the two groups (57.2 vs 56.0y old respectively). In the second explant culture study (*Exp. II*, Supplementary Table 1), of the 11 subjects, 6 subjects had BMI < 30 with an average of 24.8 kg/m^2^ (range 23–27), and 5 subjects had BMI ≥ 30 with an average of 36.3 kg/m^2^ (range 30–50). There was also no difference in age.

### Obesity created an inflammatory microenvironment in mammary tissue

**Figure 1:**
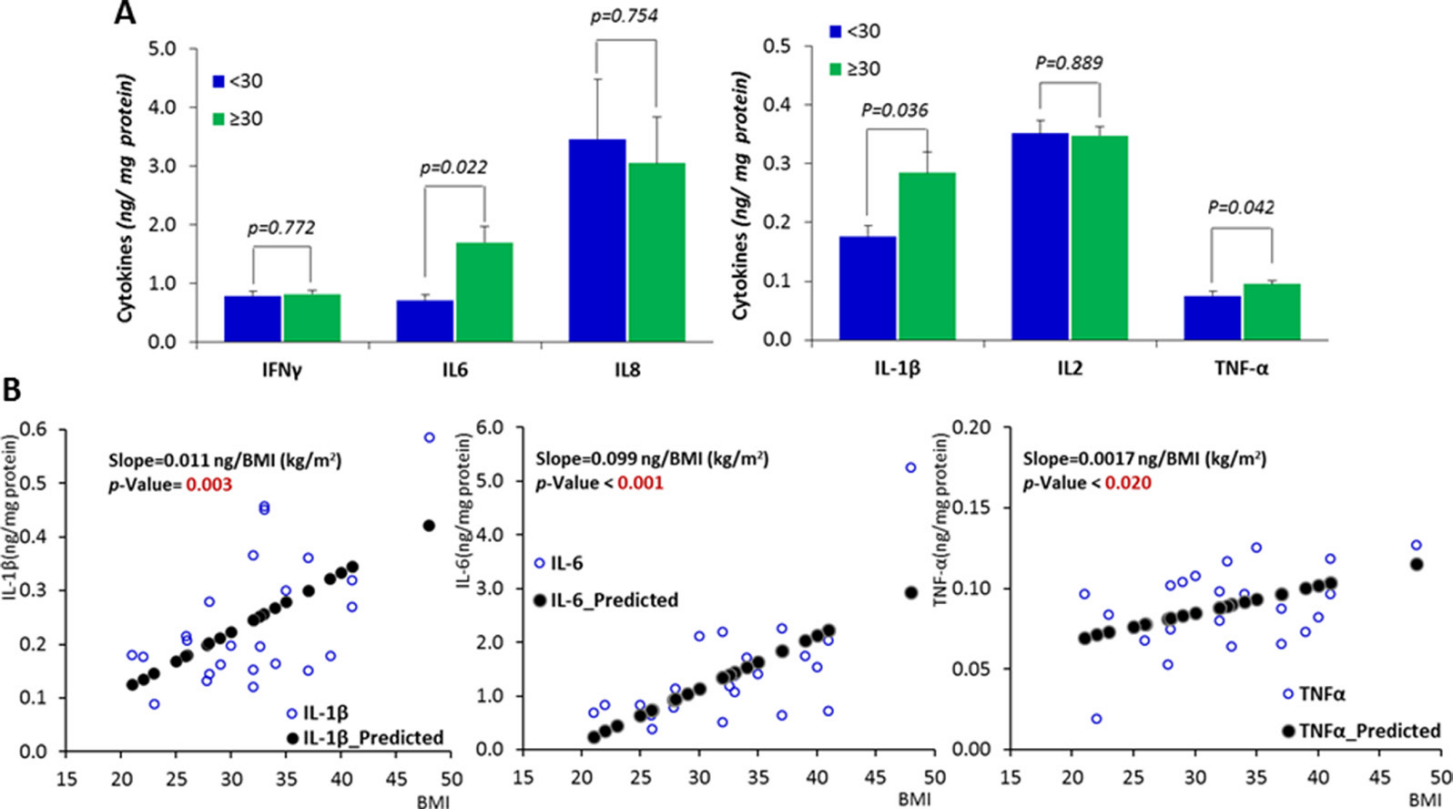
Inflammatory status in the mammary tissue of women with different BMIs (**A**) Comparisons between obese subjects (BMI ≥ 30 and subjects with BMI < 30. (**B**) The correlations between the expression of inflammatory cytokines and BMI. Data are represented as mean ± SEM.

To evaluate the inflammatory state in the breast tissue from obese and non-obese women, we measured six pro-inflammatory cytokines (IFN-γ, IL-1β, IL-2, IL-6, IL-8 and TNF-α) for those 26 tissue homogenates in *Exp. I*, significantly higher concentrations (*p* < 0.05) of IL-1β, IL-6 and TNF-α were found in breast tissue samples from obese women (Figure 1A). Linear regression between BMI and those cytokines indicates that, for every 5 (kg/m^2^) units increase in BMI, the protein level of IL-1β, IL-6 and TNF-a was significantly increased by 0.055 (*p* = 0.003), 0.495 (*p* < 0.001) and 0.0085 (*p* = 0.020) ng/mg of those cytokines, respectively (Figure 1B).

### Influence of obesity on the expression of genes along the *Wnt*-signaling cascade

**Figure 2:**
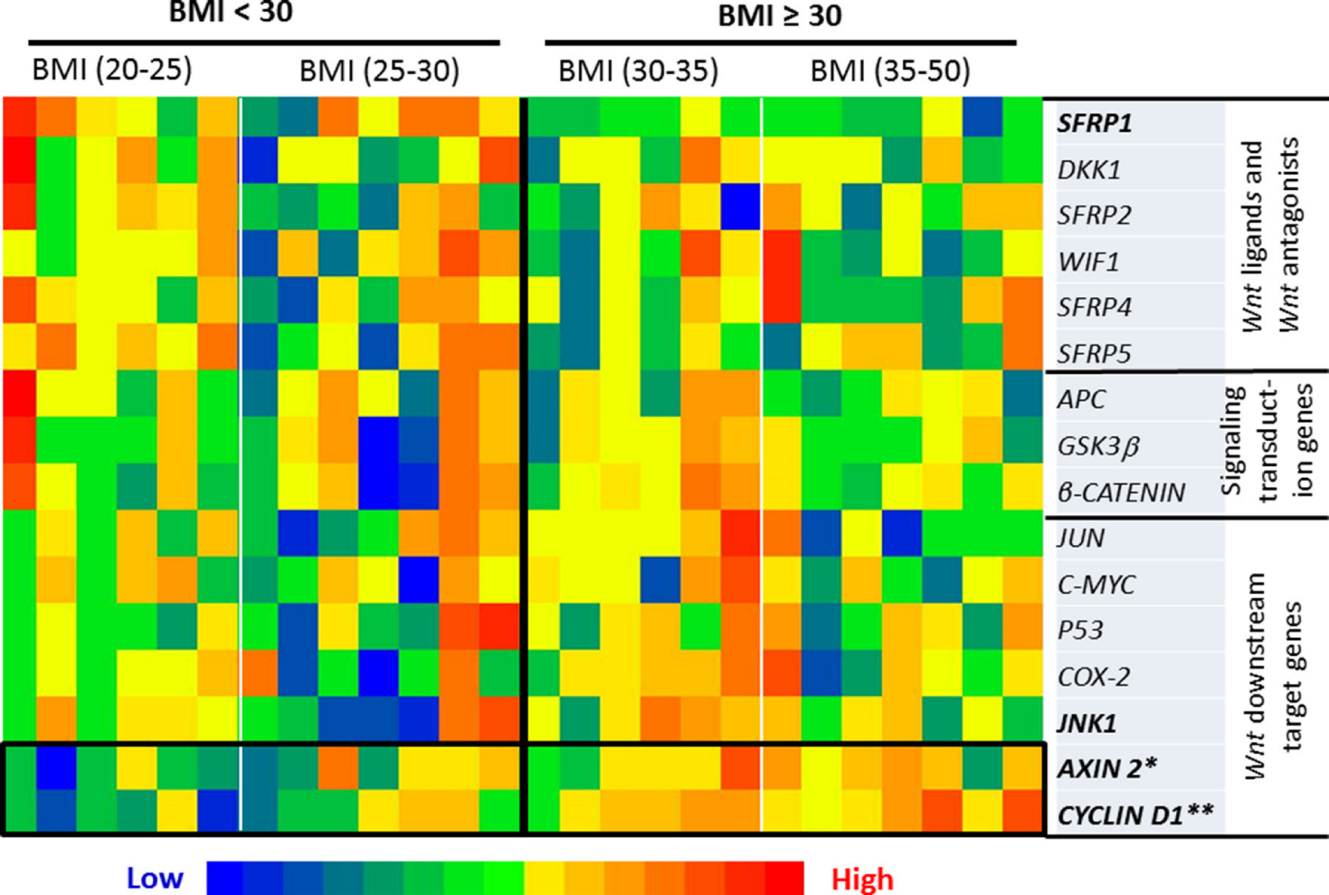
Heatmap of the transcriptional expression of *Wnt* pathway-specific genes When a comparison was made between the subjects with BMI < 30 vs BMI ≥ 30, the expression was significantly up-regulated for *CYCLIN D1 (p < 0.01)* and *AXIN 2 (p < 0.05)*, with marginal decrease of the expression for *SFRP1 (p = 0.0578)* and increase for JNK1 *(p = 0.0582)* for the individuals with BMI ^3^ 30. Significance was accepted when *p* < 0.05 with a False Discovery Rate cutoff of q ≤ 0.25 applied for multiple comparison.

**Figure 3:**
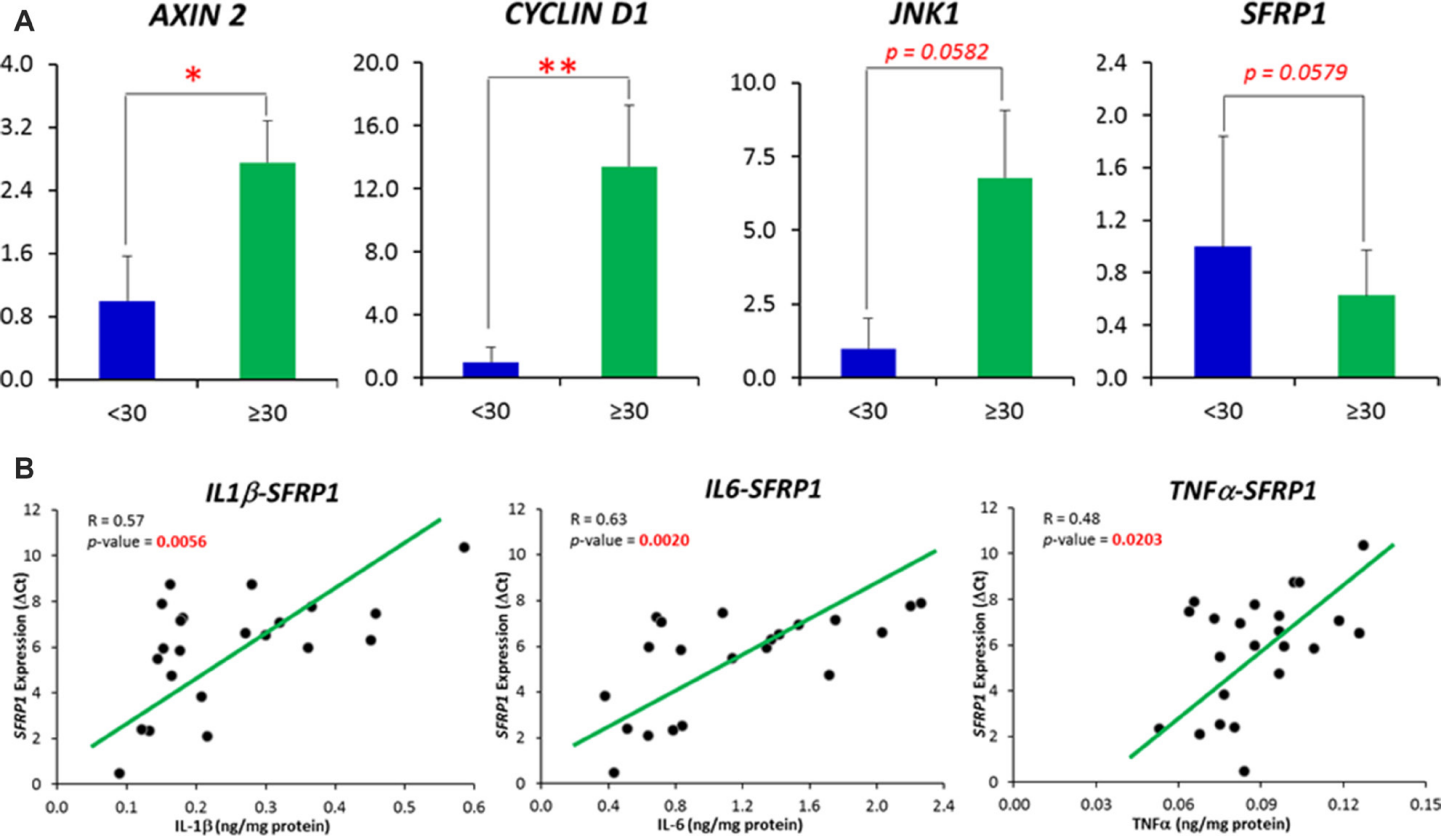
(**A**) Comparisons of *Wnt* pathway specific-genes whose expression was demonstrated to be significantly and marginally different between obese subjects (BMI ≥ 30) and individuals with BMI < 30. (**B**) Correlations between inflammatory cytokines (IL1β, IL6 and TNFα) with the expression of *SFRP1* gene. Correlations were displayed between those inflammatory cytokines and the DCt of *SFRP1* gene. The ΔCt, other than the relative expression, follows a normal distribution. A high ΔCt indicates a low expression of the gene. Data are represented as mean ± SEM.

The expression of each gene from the the *Wnt*-signaling cascade, including 6 *Wnt* ligands and *Wnt* antagonists, 3 signaling transduction genes, and 7 *Wnt* downstream target genes, was measured in the 26 samples in *Exp. I* (Figure 2). Of these 16 genes along the *Wnt*-signaling cascade, when a comparison was made between the subjects with BMI < 30 vs BMI ≥ 30, the expression was significantly up-regulated for *CYCLIN D1 (p < 0.01)* and *AXIN2 (p < 0.05)*, with marginal decrease of the expression for *SFRP1 (p = 0.0578)* and increase for *JNK1 (p = 0.0582)* for the individuals with BMI ≥ 30 (Figure 3A).

When Pearson's correlation analyses were performed between IL-1β, IL-6 and TNF-α, the cytokines whose concentrations were identified to be altered in an obese state and those *Wnt* pathway specific-genes whose expression were significantly or marginally different between obese individuals and those with BMI < 30, we observed that all 3 inflammatory cytokines were negatively associated (*p* < 0.05) with the expression *SFRP1*, as indicated by an increased DCt (Figure 3B). In addition, the expression of *CYCLIN D1* and *JNK1* was positively associated with IL-6 and TNF-α respectively (Data not shown).

### The regulation of expression of *Wnt* pathway downstream genes by treatment with anti-TNF-α antibody or TNF-α recombinant proteins

**Figure 4:**
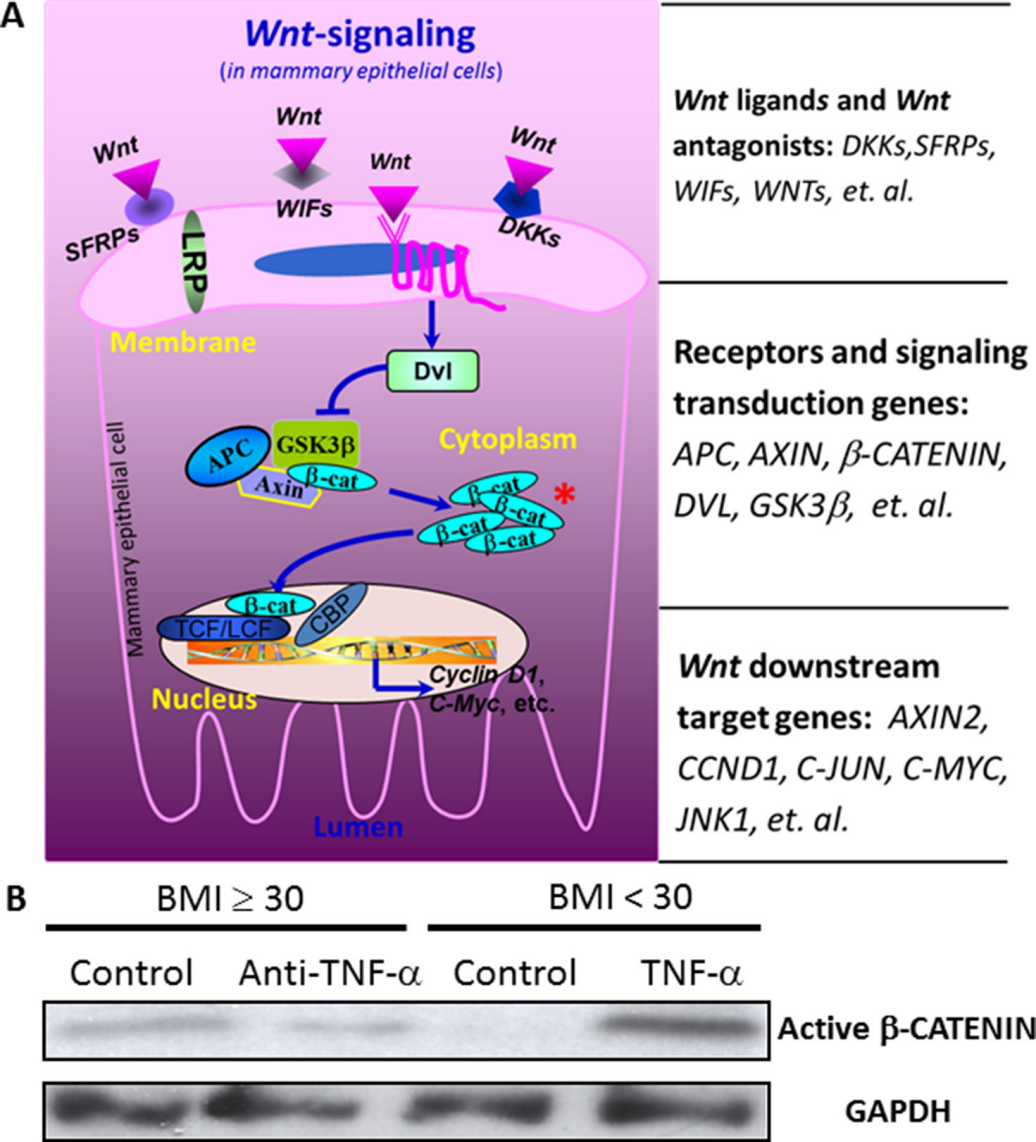
The influences of TNF-α on the expression of *Wnt* pathway downstream genes The treatment of anti-TNF-α antibody for the mammary tissue from obese subjects significantly diminished the expression of *CYCLIN D1* and *COX2* (*p* < 0.05), whereas the treatment with TNF-α recombinant protein for the breast tissue from individuals with BMI < 30 significantly increased *CYCLIN D1* expression (*p* < 0.05), but decreased *P53* expression (*p* < 0.05). A high DCt indicates a low expression of the gene. The values in the parentheses of the sample ID are BMIs for the subjects from whom the samples were collected.

To evaluate whether there is a causal relationship between elevated inflammatory cytokines and *Wnt*-signaling, in the explant study (*Exp. II*), we treated fresh mammary tissues with anti-TNF-α antibody or TNF-α recombinant proteins. We assumed mammary tissue from obese women might have an elevated level of inflammation, whereas mammary tissue from women with BMI < 30 might have a low level of inflammation. Therefore, we treated samples from obese women with anti-TNF-α antibody but treated samples from women with BMI < 30 with TNF-a recombinant proteins, with the intention to detect the impact of TNF-α, which otherwise might not be detected if opposite treatments were applied. The expression of *Wnt*-signaling pathway downstream genes was subsequently meaured. We found that treatment with anti-TNF-α antibody for those mammary tissues from women with BMI ≥ 30 diminished the expression of *CYCLIN D1* and *COX2* (*p* < 0.05) in obese individuals, whereas treatment with TNF-α recombinant protein in samples from individuals with BMI < 30 significantly increased *CYCLIN D1* expression (*p* < 0.05) and marginally increased *AXIN2* expression (*p* = 0.082), but decreased *P53* expression (*p* < 0.05) (Supplementary Table 2). The change in the expression of *Wnt* target genes in individuals under each treatment condition is shown on Figure 4 and the detailed relative expression and significances are shown in Supplementary Table 2. Note that, in Figure 4, a high DCt indicates a low expression of the gene.

### The treatment with TNF-α recombinant proteins TNF-α increased, whereas the treatment with anti-TNF-α antibody decreased the accumulation of active β-CATENIN

**Figure 5:**
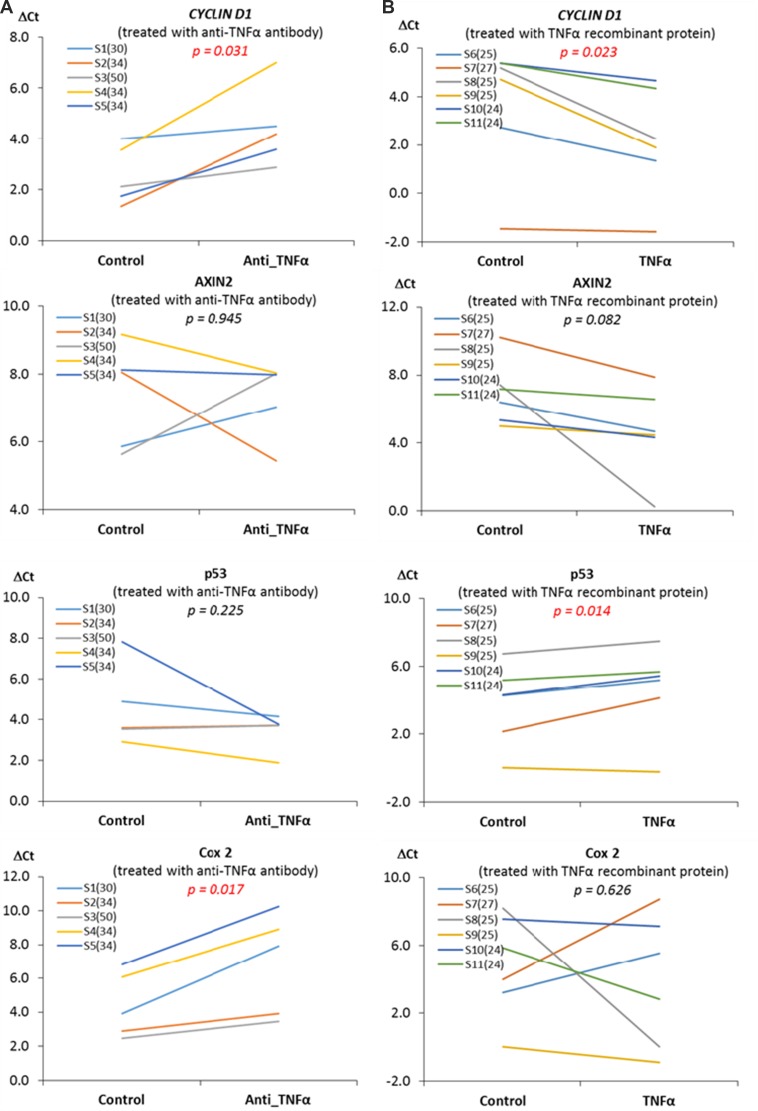
The activation of *Wnt*-signaling pathway by TNF-α (**A**) *Wnt*-signaling pathway. A key indicator of the activation of the canonical *Wnt*-signaling pathway is the accumulation of active β-CATENIN. (**B**) The influences of TNFα on β-CATENIN. The explant culture of mammary tissue from obese women with anti-TNF-α antibody decreased the level of active β-CATENIN (the dephosphorylated form), whereas the culture with TNF-α recombinant protein for the mammary tissue from individuals with BMI < 30 increased the level of active β-CATENIN.

After observed that treatment with anti-TNF-α antibody or TNF-α recombinant proteins altered expression of *Wnt* pathway downstream genes, which collectively indicates the elevation of *Wnt*-signaling by TNF-α, we further examined the accumulation of active β-CATENIN, a key indicator of the activation of the canonical *Wnt*-signaling pathway (Figure 5A). The explant culture of mammary tissue from obese women with anti-TNF-α antibody decreased the level of active β-CATENIN (the dephosphorylated form), whereas the culture with TNF-a recombinant protein for the mammary tissue from individuals with BMI < 30 increased the level of active β-CATENIN (Figure 5B).

## DISCUSSION

Obesity is an established risk factor for postmenopausal breast cancer as reported by systematic reviews [1] and meta-analyses [2] which summarized a large body of observational studies. As the prevalence of obesity has increased by an alarming rate in the United States, and a further increase is predicted to occur over the next two decades [20, 21], it is important to understand the pathophysiological mechanism driving the association between obesity and the development of postmenopausal breast cancer. Although estrogen has been suggested as a mechanism responsible for the association between obesity and postmenopausal breast cancer [22], there is substantial evidence that other factors, such as pro-inflammatory cytokines and insulin-like growth factors, may suppress programmed cell death and thereby favor breast carcinogenesis [23]. Findings from this study demonstrate that inflammatory cytokines in the mammary tissue were associated with altered expression of *Wnt* pathway specific-genes. The data from the *ex vivo* treatment with anti-TNF-α antibody or TNF-α recombinant protein further demonstrated TNF-α possesses the role to drive the elevation of *Wnt*-signaling. These results collectively suggest that the inflammation-driven activation of *Wnt* pathway represents a mechanism by which obesity promotes breast tumorigenesis in postmenopausal women.

The expansion of adipose tissue observed in obesity is accompanied by an increase in the secretion of pro-inflammatory cytokines by the adipocytes, pre-adipocytes and macrophages that infiltrate the adipose tissue. These cytokines secreted from peripheral adipose tissue can enter the circulation and migrate to other tissues including the breast, and in fact elevated cytokine levels have been found in breast biopsies from cancer patients [24]. Enlarged or necrotic adipocytes in the breast of obese individuals can stimulate infiltration of macrophages, which can also produce a pro-inflammatory microenvironment locally in mammary tissue [25, 26]. In the present study, we have clearly demonstrated that obesity is associated with an elevated inflammatory status in breast tissue, as indicated by augmented levels of inflammatory cytokines in mammary tissue. Of the 6 examined cytokines (IFN-γ, IL-1β, IL-2, IL-6, IL-8 and TNF-α), we observed significant increases for IL-1β, IL-6 and TNF-α in obese subjects when compared to individuals with BMI < 30. Previous studies have also demonstrated elevated cytokine levels in primary human breast cancers, such as IL-6 [27] and IL-8 [28]. However, to date, there are no well-established mechanistic connections between these increased mammary cytokines in obese individuals and the development of breast cancer. This study explored the role of mammary pro-inflammatory cytokines in the up-regulation of the *Wnt*-signaling. Alterations in the *Wnt* pathway are present in ~60% of breast cancers [16, 17], and a recent study showed that 99% of breast tumor samples (156/158) analyzed in the study had alterations in at least one gene within the *Wnt* pathway cascade, indicating the importance of this pathway in the development of this breast cancer [29]. We observed that the expression of *CYCLIN D1* and *AXIN2* were significantly upregulated in obese subjects (*p* < 0.05) with a tendency for upregulation of *JNK1* (*p* = 0.0582). We also observed that the expression of *SFRP1* was marginally increased (*p* = 0.0579) in obese subjects, but it significantly correlated (*p* < 0.05), in a negative fashion, with all 3 inflammatory cytokines, IL-1β, IL-6 and TNF-α, which were identified to be significantly increased in an obese state. *C-MYC* and *CYCLIN D1* are generally considered as critical *Wnt* pathway downstream genes. We did not see alterations in *C-MYC* expression, but it is not surprising since *C-MYC* is also well-known to be influenced by a number of factors other than *Wnt*-signaling. For instance, *C-MYC* has been proposed as a putative target gene of signal transducer and activator of transcription 5 (*STAT5*) [30]. *AXIN2* is considered as a more direct target of the *Wnt* pathway and it is therefore a common readout for *Wnt*-signaling activation [31, 32]. In overall, the data of the expression profile of *Wnt*-signaling target genes indicated its elevation in obese subjects in response to obesity-driven inflammation (Figure 2).

To define the causal relationship between inflammatory cytokines and the regulation of *Wnt*-signaling targets in mammary tissue, we examined one of the most critical inflammatory cytokines, TNF-α, a cytokine consistently elevated in obese individuals and also a known activator of the *Wnt* pathway [18, 19]. In the explant culture study, we treated mammary tissues from obese women, which have a high level of TNF-α, with a neutralizing anti-TNF-α antibody, whereas treated breast tissues from individuals with BMI < 30, which may have a relatively low level of TNF-α, with TNF-α recombinant protein. Our results clearly demonstrated that there was a downregulation of *Wnt*-signaling by anti-TNF-α antibody, but an upregulation of *Wnt*-signaling by TNF-α recombinant protein, as indicated the expression of *Wnt* pathway downstream genes and the accumulation of β-CATENIN (Figures 4 and 5).

Although our findings suggest that TNF-α is a potent activator of *Wnt* signaling, work from other groups showed that there are potential paradoxical roles of TNF-α in terms of the development of cancer. Balkwill reported that TNF-α, when administered at supra-physiological levels, has powerful anti-cancer actions, but has tumor-promoting effects when chronically produced in the tumor microenvironment [33]. Another study showed that elevated serum TNF-α (> 6.20 pg/mL) was associated with a 52% decreased risk of progression of breast cancer [34], whereas other studies have shown that blockade of TNF-α inhibits cell proliferation and induces apoptosis in a triple negative breast cancer cell line [35], and its deletion is also able to inhibit migration, invasion and metastasis [36]. In the context of obesity, where TNF-α is chronically elevated, TNF-α may play a pro-carcinogenic effect. Results from the present study are consistent with the latter: obesity-induced elevation of TNF-α may increase susceptibility to tumorigenesis via the activation of the *Wnt*-signaling in mammary tissue.

It is noteworthy that, in the present study, we also observed elevated levels of two other pro-inflammatory cytokines, IL-1β and IL-6, in the mammary tissues from obese women. These cytokines may also play a role in regulating the *Wnt*-signaling pathway. For instance, in the examination of the effect of inflammation on 3T3-L1 preadipocyte development and differentiation to mature adipose cells, Gustafson and Smith demonstrated that both IL-6 and TNF-α altered components in the canonical *Wnt* pathway cascade, such as *AXIN2*, *DVL*, and β*-CATENIN* levels [37]. Similar to TNF-α [18, 19], IL-β from macrophages could also induce phosphorylation of GSK3β, stabilize β-CATENIN, and thereby increase the expression of *Wnt* pathway downstream target genes in tumor cells [38, 39].

In summary, the present study showed that obesity created a low-grade chronic inflammatory microenvironment in the mammary tissue of women, as indicated by the elevations of several inflammatory cytokines, including TNF-α, IL-6, and IL-1β. These cytokines were associated with altered expression of several genes within the *Wnt*-signaling cascade, which are in a pattern indicating its activation. The *ex vivo* culture with anti-TNF-α antibody or TNF-α recombinant protein indicated a causal role of TNF-α in the regulation of the *Wnt* pathway. These findings collectively demonstrate that obesity creates a chronic inflammatory microenvironment in mammary tissue, and the elevated pro-inflammatory cytokines may drive the elevation of *Wnt*-signaling.

## MATERIALS AND METHODS

### Patients for breast tissue samples

Study subjects were women who underwent reduction mammoplasty at Baystate Medical Center in Springfield, Massachusetts, USA, and the protocol was approved by the Institutional Review Board at Baystate Medical Center. All participants consented to provide excess tissues from the breast not needed for diagnostic purposes. Anthropometric characteristics of the subjects are shown in Supplementary Table 1.

In the first association study (*Exp. I*), we examined the association between inflammatory cytokines and the expression of *Wnt*-signaling target genes in mammary tissue from women with age ≥ 50y and BMIs ranging from 21 to 48. Samples were selected from the tissue bank at the Biospecimen Resource and Molecular Analysis Facility in the Baystate Medical Center. The inflammatory cytokine profile and the expression of *Wnt*-signaling targets were measured using an electrochemiluminescence assay and real-time PCR respectively.

In the second *ex vivo* tissue culture study (*Exp. II*), we investigated the causal role of TNF-α in the regulation of *Wnt*-signaling using an explant culture of fresh mammary tissue collected from women who underwent elective breast reduction surgery. Mammary tissue was harvested and immediately minced and placed on Surgifoam gelatin sponges (Ferrosan, Sueborg, Denmark) in 60 mm tissue culture dishes containing 2 mL of whole organ culture medium and maintained for 24 h in 5% CO_2_. Tissues obtained from obese women (BMI < 30) were treated with 2.5 μg/ml anti-TNF-a antibody (Abcam, Cambridge, MA), whereas tissues from women with BMI < 30 were treated with TNF-α recombinant protein (Sigma, Saint Louis, MO). Explant tissue was then flash frozen in liquid nitrogen and stored at −80°C for subsequent real-time PCR assay for *Wnt* pathway targets.

### Pro-inflammatory cytokines profile

A total of 26 mammary tissue samples (*Exp. I*) were collected and homogenized in a lysis buffer (10-20 mg of tissue per 400 μL of buffer) with phosphatase and protease inhibitors (Sigma, St. Louis, MO) added to inhibit degradation. The lysis buffer was prepared according to the protocol for the electrochemiluminescence assay (Meso Scale Discovery, Rockville, MD). The lysate was made from a Tris lysis buffer consisting of 150 mM NaCl, 20 mM Tris, 1 mM EDTA, 1 mM EGTA and 1% Triton-X-100 with pH adjusted to 7.5. Samples were homogenized and centrifuged at 4°C at 12,000 rpm for 30min, and then the protein fraction was collected. The total protein concentrations were determined using a commercially available Pierce^TM^ BCA Protein Assay Kit (Thermo Fisher Scientific, Waltham, MA) and the SpectraMax microplate reader (Molecular Devices, Sunnyvale, CA). Six inflammatory cytokines, IL-1β, IL-2, IL-6, IL8, IFNγ and TNF-α, were measured using the QuickPlex SQ 120 imager (Meso Scale Discovery, Rockville, MD). Assays were performed according to the manufacturer's instructions and cytokines are expressed as ng of cytokine per milligram of total protein. All standards and samples were measured in duplicate.

### *Wnt*-signaling pathway specific gene expression profile

The relative expression was determined 16 genes selected from the Wnt pathway cascade including genes of Wnt ligands and Wnt antagonists, signaling transduction genes, and the downstream target genes. Briefly, total RNA was extracted from the small intestine with Trizol (Invitrogen, Carlsbad, CA), and cDNA was synthesized with SuperScript III (Invitrogen, Carlsbad, CA, USA). Real-time PCR was performed on the ViiA^™^ 7 Real-Time PCR System (Applied Biosystems, Foster City, CA). Primer sequences were listed in Supplementary Data (Supplementary Table 3).

### Western blot for active β-CATENIN

For immunoblot, protein were run on a SDS-PAGE gel and transferred onto PVDF membranes, which were then incubated with specific primary antibodies followed by secondary antibodies for detection. Anti-Active-β-catenin was purchased from EMD Millipore (Billerica, MA). The epitope corresponds to amino acid residues 36–44 of β-catenin antibody, and specifically recognizes active form of β-catenin with dephosphorylation on Ser37 or Thr41.

### Data analysis

Data are expressed as means ± SEM, and statistical analysis was performed using SAS (Version 9.4, SAS Institute, Cary, NC). Because the population of women undergoing reduction mammoplasty typically have high BMIs, we combined the normal (BMIs between 18–25) and the overweight (BMIs between 25–30) women together and grouped subjects into two categories: samples from individuals with a BMI < 30 or BMI ≥ 30. For the data analysis in the association study (Exp. I), a linear regression analysis was performed to examine the production of inflammatory cytokines in response to BMI. For the Wnt pathway gene expression profile, significance was accepted when *p* < 0.05 with a False Discovery Rate (FDR) cutoff of *q* < 0.25 applied. Pearson's correlation was performed to determine the association between expression of the Wnt pathway specific genes and inflammatory cytokines. For the analysis of the explant culture study (Exp. 2), a paired *T-test* was used, and also when a multiple comparison was performed, significance was accepted with *p* < 0.05 and a FDR cutoff of *q* < 0.25. The expression of each gene was normalized to the housekeeping gene GAPDH (ΔCt = Ct_target gene_-Ct_GAPDH_). Statistical analyses were performed based on differences in ΔCt between individuals with BMI < 30 and individuals with BMI ≥ 30 and relative expression is reported as 2^−ΔΔCt^, where ΔΔCt = ΔCt_BMI≥30_−ΔCt_BMI<30_.

## SUPPLEMENTARY MATERIALS TABLES


