# Supplementary material for: The influence of tumor necrosis factor-α on the tumorigenic Wnt-signaling pathway in human mammary tissue from obese women
Source: Oncotarget. 2017 Mar 28;8(22):36127–36. doi: 10.18632/oncotarget.16632 (PMC5482643; doi:10.18632/oncotarget.16632)
Supplement: Supplementary file 1 [file oncotarget-08-36127-s001.pdf]

The influence of tumor necrosis factor-α on the tumorigenic *Wnt*-signaling pathway in human mammary tissue from obese women

Supplementary Materials

Supplementary Table 1: Anthropometric characteristics of the subjects

Categories		Age (year)		BMI (kg/m^2)	
**BMI**	***n***	**Mean**	**Range**	**Mean**	**Range**
Experiment I (n=26)					
<30	10	57.2 ± 1.4	51–65	25.6 ± 0.9[a]	21–29
≥30	16	56.0 ± 1.8	51–66	36.0 ± 1.2[b]	30–48
Experiment II (explant culture)					
< 30	6	53.1 ± 1.4	50–60	24.8 ± 0.4[a]	23–27
≥ 30	5	53.8 ± 3.4	50–67	36.3 ± 3.5[b]	30–50

[a,b]denote significant differences among the two BMI categories ($p < 0.05$).

Supplementary Table 2: Relative expressions of *Wnt* pathway targeting genes between the untreated and treated mammary tissues with anti-TNF-α antibody or TNF-α recombinant protein

Wnt-signaling Targets	Anti-TNF-α Antibody[1]	*p*-Value	TNF-α Recombinant Protein[2]	*p*-Value
CYCLIN D1	0.36 ± 0.12	**0.031**	3.69 ± 1.18	**0.023**
C-MYC	0.76 ± 0.30	0.448	1.02 ± 0.28	0.836
AXIN2	2.01±1.08	0.945	2.67 ± 0.69	0.082
P53	4.52 ± 3.14	0.225	0.51 ± 0.08	**0.014**
COX2	0.26 ± 0.10	**0.017**	2.46 ± 1.96	0.626
JNK1	0.78 ± 0.60	0.323	2.00 ± 0.25	0.115
JUN	2.39 ± 0.89	0.703	7.38 ± 4.12	0.464

[1]Samples from women with BMI ≥ 30 were treated with anti-TNF-α antibody. The relative expression was determined by paired *T*-Tests between the treated and untreated samples from the same individuals. Significance was accepted when $p < 0.05$ with a False Discovery Rate cutoff of $q \leq 0.25$ applied.

[2]Samples from women with BMI < 30 were treated with TNF-α recombinant protein, and the same paired *T*-tests were used. Significance was accepted when $p < 0.05$ with a False Discovery Rate cutoff of $q \leq 0.25$ applied.

Supplementary Table 3: Primers and relative expressions of genes within the mammary *Wnt* pathway cascade when comparing individuals with BMI < 30 with obese individuals (BMI ≥ 30)

***Wnt* Pathway Cascade**	**Genes**	**Forward Primer**	**Reverse Primer**	**BMI < 30**	**BMI ≥ 30**	***P* value***
***Wnt* Ligands & *Wnt* Antagonists**	*DKK1*	CCTT GAAC TCGG TTCT CAAT TCC	CAAT GGTC TGGT ACTT ATTC CCG	1.00 ± 2.02	6.17 ± 3.61	0.965
	SFRP1	ACGT GGGC TACA AGAA GATG G	CAGC GACA CGGG TAGA TGC	1.00 ± 0.84	0.63 ± 0.34	0.058
	SFRP2	TTGCTCTTTGTCTCCAGGATG	CGACATCATGGAAACCCTTT	1.00 ± 0.99	2.61 ± 0.88	0.707
	SFRP4	ATCATCCTTGAACGCCACTC	TCGAACACAAGTCCCTCTCA	1.00 ± 0.72	1.91 ± 0.77	0.937
	SFRP5	TGTGCTCCATCTCACACTGG	CTGGACAACGACCTCTGCAT	1.00 ± 0.76	0.82 ± 0.24	0.849
	WIF1	TCTC CAAA CACC TCAA AATG GT	GACA CTCG CAGA TGCG TCT	1.00 ± 1.17	7.23 ± 5.21	0.714
**Receptors & Signaling Transduction Genes**	*APC*	GGAC CGGG AACG GTGT TTG	AGCT GAGA GTAG TACC AGAG C	1.00 ± 0.49	2.21 ± 0.75	0.982
	GSK3B	GGCA GCAT GAAA GTTA GCAG A	GGCG ACCA GTTC TCCT GAAT C	1.00 ± 1.16	4.87 ± 1.58	0.391
	β-CATENIN	AAAG CGGC TGTT AGTC ACTG G	CGAG TCAT TGCA TACT GTCC AT	1.00 ± 1.43	6.67 ± 2.24	0.168
***Wnt*-Signaling Target Genes**	*AXIN2*	CAAC ACCA GGCG GAAC GAA	GCCC AATA AGGA GTGT AAGG ACT	1.00 ± 0.56	2.76 ± 0.53	0.030
	C-MYC	ATGG CCCA TTAC AAAG CCG	TTTC TGGA GTAG CAGC TCCT AA	1.00 ± 0.85	5.75 ± 2.64	0.524
	COX2	CTGG CGCT CAGC CATA CAC	CGCA CTTA TACT GGTC AAAT CCC	1.00 ± 1.71	5.18 ± 1.66	0.307
	CYCLIN D1	GCTG CGAA GTGG AAAC CATC	CCTC CTTC TGCA CACA TTTG AA	1.00 ± 0.95	13.40 ± 3.85	< 0.01
	JNK1	TGTG TGGA ATCA AGCA CCTT C	AGGC CTCA TCAT AAAA CTCG TTC	1.00 ± 1.01	6.78 ± 2.29	0.058
	JUN	TCCA AGTG CCGA AAAA GGAA G	CGAG TTCT GAGC TTTC AAGG T	1.00 ± 0.38	2.06 ± 0.57	0.536
	P53	CAGC ACAT GACG GAGG TTGT	TCAT CCAA ATAC TCCA CACG C	1.00 ± 0.38	8.11 ± 2.98	0.065
GAPDH		GGAG CGAG ATCC CTCC AAAA T	GGCT GTTG TCAT ACTT CTCA TGG	N/A	N/A	N/A

*The statistical analysis is based on the ΔCt value. Significance was accepted when $p < 0.05$ with a False Discovery Rate cutoff of $q \leq 0.25$ applied for multiple comparison.
